# The Association of Depression with Obstructive Sleep Apnea in Patients with Cystic and Non-Cystic Fibrosis Bronchiectasis

**DOI:** 10.3390/life14081026

**Published:** 2024-08-19

**Authors:** Baran Balcan, Duygu Vezir, Sehnaz Olgun Yildizeli, Derya Kocakaya, Berrin Ceyhan

**Affiliations:** 1 Department of Pulmonary Medicine, Faculty of Medicine, Koc University, Istanbul 34460, Turkey; 2 Department of Pulmonary Medicine, Sureyyapasa Teaching and Researh Hospital, Istanbul 34844, Turkey; duyguvezir01@gmail.com; 3 Department of Pulmonary Medicine and Intensive Care, Faculty of Medicine, Marmara University, Istanbul 34722, Turkey; drsehnazolgun@yahoo.com (S.O.Y.); drderyagun@gmail.com (D.K.); ceyhan.berrin@gmail.com (B.C.)

**Keywords:** obstructive sleep apnea, cystic fibrosis, depression, quality of life, polysomnography, Epworth Sleepiness Scale

## Abstract

Obstructive sleep apnea (OSA) and cystic fibrosis (CF) are chronic conditions that profoundly impact quality of life. OSA, characterized by repeated episodes of upper airway collapse, can exacerbate CF symptoms due to nocturnal airway obstruction. Recent studies highlight the prevalence of OSA in CF patients, especially in adults, and its detrimental effects on health and quality of life. From April 2019 to December 2021, we conducted a study with 104 bronchiectasis patients at Marmara University Pendik Training and Research Hospital. After exclusions, 70 participants (35 CF and 35 non-CF) were included. Sleep parameters were assessed with polysomnography, and depressive mood was evaluated using the Zung Self-Rating Depression Scale (SDS). Daytime sleepiness was measured using the Epworth Sleepiness Scale (ESS). The statistical analyses included t-tests, chi-square tests, and logistic regression. Among the CF patients, depressive mood was significantly associated with female sex (OR: 4.28, 95% CI: 1.27–12.04) and anemia (OR: 7.87, 95% CI: 1.50–41.27). Higher ESS scores indicated greater daytime sleepiness in the depressive groups (*p* = 0.051). Depressive CF patients also had a significantly longer disease duration and more frequent annual exacerbations. No significant differences were found in total sleep time, sleep efficiency, or sleep stages between the depressive and non-depressive groups. A lower forced vital capacity (FVC) was observed in the depressive CF patients, although not significantly. Depression is prevalent among adult CF patients with OSA, with significant associations with female sex and anemia. These findings underscore the need for integrated care addressing both physical and mental health aspects, including interventions for respiratory symptoms, anemia management, and sleep quality enhancement to improve overall quality of life.

## 1. Introduction

OSA is a chronic condition characterized by repetitive episodes of upper airway collapse, leading to blood oxygen desaturation and symptoms like excessive daytime sleepiness (EDS) [1].

Bronchiectasis is a chronic lung disease marked by the irreversible dilatation of the bronchi, [2,3,4] CF, primarily caused by a mutation in the cystic fibrosis transmembrane regulator (CFTR) gene affecting the chloride channels, often leads to recurrent lower respiratory tract infections and sputum accumulation, potentially causing nocturnal airway obstruction and OSA [4]. 

Notably, mild OSA may even manifest as an initial symptom of CF in children [5]. Recent studies have shed light on the prevalence and impact of OSA in CF patients, revealing its commonality and significant adverse effects on quality of life, especially in adults, a demographic previously understudied compared to children [6,7]. Alarmingly, OSA affects 10–40% of adults with CF, significantly impacting their health and quality of life [8]. CF and OSA are chronic conditions that significantly affect the quality of life and mental health of those diagnosed. 

Recent studies have begun to uncover the intricate relationship between these conditions, focusing on their impact on sleep quality, daytime sleepiness, and overall health-related quality of life [9]. This study aims to delineate the prevalence and implications of depression and quality of life in adult CF patients suffering from OSA.

## 2. Materials and Methods

**Figure 1:**
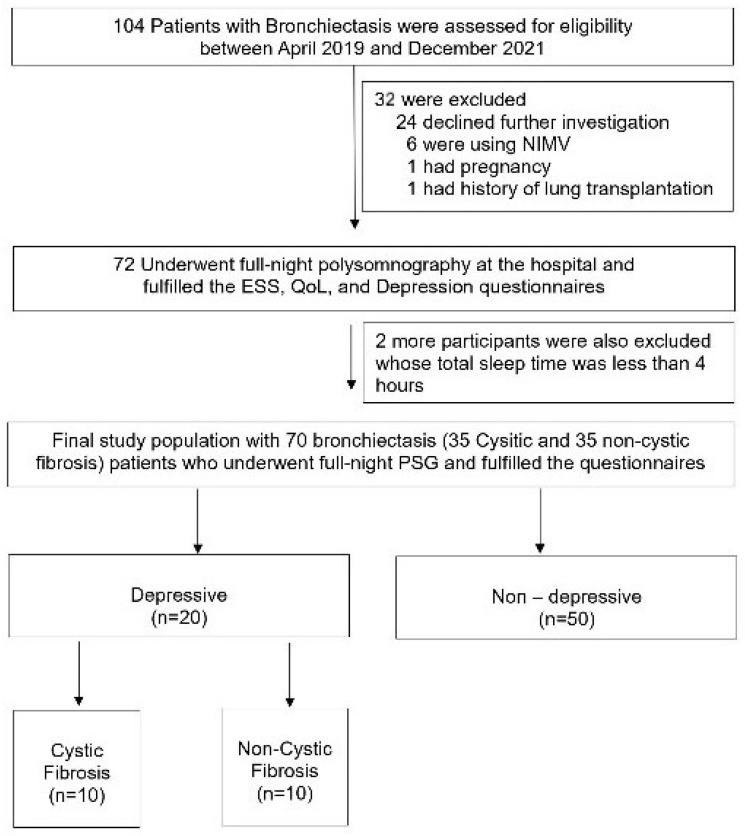
Flowchart of the participants.

The study, conducted from April 2019 to December 2021, involved 104 adult bronchiectasis patients from the Chest Diseases outpatient clinic at Marmara University Pendik Training and Research Hospital, Istanbul, Turkey. As depicted in Figure 1, all 104 patients initially met the eligibility criteria. However, after excluding 34 individuals for various reasons, 35 CF and 35 non-CF patients were ultimately included in the main study protocol. Informed consent was obtained from each participant. Additionally, the study protocol received ethical approval from the Ethical Committee of Marmara University, with the approval number being 09.2019.503. 

### 2.1. Polysomnography

In the sleep studies, the Embletta (Embla, Broomfield, CO, USA) device was employed to record various sleep parameters. Sleep stages were monitored through electroencephalography (EEG), eye movements via electrooculography (EOG), and leg movements by electromyography (EMG). To measure nasal pressure, a nasal cannula or pressure transducer system was utilized, and to detect body position, as well as thoracic and abdominal movements, plethysmography belts were used. Heart rate and blood oxygen saturation were determined using a finger pulse oximeter. We excluded participants with a total sleep time of less than four hours. Apnea and hypopnea were defined following the latest American Academy of Sleep Medicine guidelines: apnea as a ≥90% cessation of airflow and hypopnea as a ≥30% reduction in nasal pressure amplitude and/or thoraco-abdominal movement for ≥10 s with significant oxyhemoglobin desaturation (decrease by at least 3% from baseline) [10]. OSA was diagnosed when the apnea–hypopnea index (AHI) was ≥5 events/h. All the recordings were analyzed by the same physician (B.B.).

### 2.2. Comorbidities

The baseline demographics, smoking habits, comorbid diseases, and comprehensive medical histories of the study participants were meticulously gathered from their medical records. Obesity was specifically defined as having a body mass index (BMI) of 30 kg/m^2^ or greater [11].

### 2.3. Group Assignment

As indicated in Figure 1, 104 adults were initially eligible for our study. After excluding a total of 34 participants, the protocol was completed with 70 patients, with 35 in each group. All the enrolled participants underwent full-night polysomnography in the hospital, and only those with a total sleep time of over 4 h were included in the final study protocol (Figure 1).

### 2.4. Epworth Sleepiness Scale

In our protocol, daytime sleepiness for all the participants was evaluated using the ESS questionnaire [12]. This tool comprises eight questions, each asking about the likelihood of dozing off in different situations over the past month. The ESS score ranges from 0 to 24, with a cut-off value of 10 used to identify patients experiencing excessive daytime sleepiness. 

### 2.5. Zung Self-Rating Depression Scale 

The evaluation of depressive mood in our study was conducted using the Turkish version of the Zung SDS at baseline. The Zung SDS is a widely recognized tool for assessing depression, offering both a total score and categorization capabilities [13]. It consists of 20 questions, each rated on a scale from 1 to 4, with 1 indicating ‘a little of the time’ and 4 ‘most of the time’. Notably, reverse scoring is applied to questions 2, 5, 6, 11, 12, 14, 16, 17, 18, and 20. All the responses were meticulously entered into our database by the same investigator (D.V.). A total score of 40 or over was accepted as indicative of depression. 

### 2.6. Pulmonary Function Testing

Each participant underwent pulmonary function testing (PFT) using a specific device (JAEGER Masterscreen, Vyaire Medical, Mettawa, IL, USA), calibrated daily by a consistent technician. The recorded metrics included forced expiratory volume in 1 s (FEV1), forced vital capacity (FVC), and forced expiratory flow between 25% and 75% of the FVC (FEF 25–75). The PFT results were interpreted as per the European Respiratory Society and American Thoracic Society guidelines [14].

### 2.7. The Modified Medical Research Council Dyspnea Scale and Charlson Scores

The Modified Medical Research Council (mMRC) dyspnea scale, originally developed by Fletcher et al. in 1940, measured participants’ perceived breathlessness. Scores range from 0 to 4, with each point denoting increasing levels of dyspnea [15]. The Charlson comorbidity index evaluated participants’ chronic diseases and associated comorbidities. This index correlates with the mortality rate observed after a 1-year follow-up [16].

### 2.8. Statistics

Data were presented as mean [standard deviation (SD)] for continuous variables with an equal distribution or as numbers (percentages) within a 95% confidence interval for categorical variables. For data with an unequal distribution, the Mann–Whitney U test was used, and values were presented as medians and interquartile ranges (IQRs). An independent samples *t*-test was used for differences between groups in means, and the chi-square test (or Fisher’s exact test when appropriate) was used to compare categorical variables. Spearman’s correlation coefficient was used to determine the relationship between parameters with an unequal distribution. The relationship between sleep apnea and all the examined parameters for the cases was evaluated using logistic regression analysis, presenting odds ratios and 95% confidence intervals. All the statistical tests were two-tailed, and a *p* value of less than 0.05 was considered significant. Statistical analysis was performed using the Statistical Package for Social Sciences, version 22.0 for Windows systems (SPSS Inc., Chicago, IL, USA).

## 3. Results

### 3.1. Demographics, Clinical Characteristics, and Sleep Parameters of All the Participants

In Table 1, there are 70 participants with bronchiectasis, divided into depressive (*n* = 20) and non-depressive (*n* = 50) groups. The average age was similar between the two groups (29.65 ± 11.50 years for depressive vs. 29.82 ± 11.94 years for non-depressive, *p* = 0.957). BMI was also comparable (22.77 ± 5.24 for depressive vs. 22.36 ± 3.78 for non-depressive, *p* = 0.750). Notably, a higher percentage of the non-depressive group was female (59.0% vs. 41.0%, *p* = 0.010). Smoking prevalence was slightly higher in the depressive group (15.0% vs. 12.0%, *p* = 0.735). The duration of disease was not significantly different between the groups (16.50 ± 8.58 years for depressive vs. 18.28 ± 8.30 years for non-depressive, *p* = 0.434). The annual exacerbation count (1.80 ± 1.44 vs. 1.58 ± 1.33, *p* = 0.542) and hospitalization count (0.85 ± 0.30 vs. 0.60 ± 0.15, *p* = 0.411) were also similar. However, the modified Medical Research Council (mMRC) score was significantly higher in the depressive group (1.60 ± 0.50 vs. 1.20 ± 0.45, *p* = 0.004), indicating worse respiratory symptoms. The Charlson comorbidity score did not differ significantly (0.35 ± 0.18 vs. 0.36 ± 0.09, *p* = 0.459). Anemia was more prevalent among the depressive group (40.0% vs. 18.0%, *p* = 0.052), although this result was marginally non-significant. Other conditions such as diabetes mellitus (10.0% vs. 16.0%, *p* = 0.517), pancreatic disease (40.0% in both groups, *p* = 1.000), and cardiac disease (5.0% vs. 2.0%, *p* = 0.496) showed no significant differences. Osteoporosis was noted in 6.0% of the non-depressive group but was absent in the depressive group (*p* = 0.263). Oxygen support was required similarly across both groups (5.0% vs. 4.0%, *p* = 0.852). The ESS scores were significantly higher in the depressive group (5.75 ± 3.65 vs. 3.72 ± 3.38, *p* = 0.030), indicating greater daytime sleepiness. Sleep-related conditions like OSA were almost equally prevalent (55.0% vs. 52.0%, *p* = 0.820). Nutritional support was more common in the depressive group (40.0% vs. 22.7%, *p* = 0.126). Lung function measures showed that the depressive group had a lower FVC (2.40 ± 1.08 L vs. 3.02 ± 0.94 L, *p* = 0.022), while other parameters like forced expiratory volume in one second (FEV1) and the FEV1/FVC ratio were not significantly different. Sleep metrics such as total sleep time, sleep efficiency, and various sleep stages did not show significant differences between the groups. The apnea–hypopnea index (AHI) and oxygen desaturation index (ODI) were also similar.

### 3.2. Demographics, Clinical Characteristics, and Sleep Parameters of the CF Patients

As illustrated in Table 2, a total of 35 cystic fibrosis patients, comprising 10 individuals with depression and 25 without depression, were evaluated for various demographic and clinical characteristics. The average age was similar between the two groups (25.90 ± 4.41 years for depressive vs. 27.04 ± 5.01 years for non-depressive, *p* = 0.276). BMI was also comparable (20.54 ± 3.32 for depressive vs. 21.75 ± 2.79 for non-depressive, *p* = 0.139). There was a higher percentage of females in the non-depressive group (60.0% vs. 40.0%, *p* = 0.084). Smoking prevalence was higher in the non-depressive group (10.0% vs. 4.0%, *p* = 0.490). Notably, the duration of disease was significantly longer in the depressive group (22.12 ± 5.43 years vs. 17.90 ± 8.53 years, *p* = 0.044). The annual exacerbation count was also significantly higher in the depressive group (2.40 ± 1.17 vs. 1.84 ± 1.14, *p* = 0.049). However, the annual hospitalization count did not differ significantly between the groups (1.10 ± 0.44 vs. 0.92 ± 0.24, *p* = 0.280). The mMRC score, which measures respiratory symptoms, was slightly higher in the depressive group (1.40 ± 0.52 vs. 1.20 ± 0.50, *p* = 0.149), though not significantly. The Charlson comorbidity score was significantly lower in the depressive group (0.00 vs. 0.32 ± 0.09, *p* = 0.001). Anemia was significantly more prevalent among the depressive group (60.0% vs. 16.0%, *p* = 0.009). Diabetes mellitus was present in 32.0% of the depressive group but absent in the non-depressive group (*p* = 0.042). Both groups had an equal prevalence of pancreatic disease (80.0%, *p* = 1.000). Osteoporosis was noted only in the non-depressive group (12.0%, *p* = 0.252). Oxygen support was required similarly across both groups (10.0% vs. 4.0%, *p* = 0.490). The ESS score was higher in the depressive group, though not significantly (6.00 ± 3.62 vs. 3.76 ± 2.85, *p* = 0.051), indicating greater daytime sleepiness. Sleep-related conditions such as OSA were more common in the depressive group (70.0% vs. 48.0%, *p* = 0.283). Nutritional support was more frequently needed in the depressive group (60.0% vs. 40.0%, *p* = 0.283). Lung function measures showed that the depressive group had a lower FVC (2.25 ± 1.17 L vs. 2.79 ± 1.02 L, *p* = 0.097), though this difference was not significant. Other lung function parameters, including FEV1 and the FEV1/FVC ratio, were not significantly different. Sleep metrics such as total sleep time, sleep efficiency, and various sleep stages did not show significant differences between the groups. The AHI and the oxygen desaturation index (ODI) were also similar. However, the average heart rate was significantly higher in the depressive group (75.57 ± 8.46 vs. 70.11 ± 8.05, *p* = 0.041).

### 3.3. Associates of Depressive Mood among CF and Non-CF Adults

The analysis of depressive mood among all the participants highlights several significant associations. In the univariate analysis, the female sex was found to have a significantly higher likelihood of experiencing depressive mood (OR: 4.69, 95% CI: 1.37–16.04, *p* = 0.014). The mMRC (Modified Medical Research Council) dyspnea scale also showed a strong association with depressive mood (OR: 5.28, 95% CI: 1.75–15.95, *p* = 0.003). Additionally, anemia was significantly associated with higher odds of depressive mood (OR: 3.04, 95% CI: 0.96–9.86, *p* = 0.048). ES indicated a slight but significant increase in the likelihood of depressive mood (OR: 1.16, 95% CI: 1.01–1.36, *p* = 0.038). Lung function, as measured by FVC, was inversely associated with depressive mood (OR: 0.49, 95% CI: 0.27–0.93, *p* = 0.028). In the multivariate analysis, the mMRC scale remained a significant predictor of depressive mood (OR: 12.43, 95% CI: 1.92–80.18, *p* = 0.008), while the association with female sex (OR: 3.27, 95% CI: 0.57–18.79, *p* = 0.183) and anemia (OR: 3.26, 95% CI: 0.65–16.26, *p* = 0.151) lost significance. Other factors such as age, BMI, smoking status, disease duration, hospitalization frequency, nutritional support, diabetes, cardiac disease, use of oxygen support, and various sleep parameters showed no significant associations with depressive mood. Similarly, no significant associations were found regarding the pulmonary function tests, including the FEV1/L, FEV1%, FVC%, and FEV1/FVC ratios (Table 3).

### 3.4. Factors Affecting Depressive Mood among CF Participants 

Among the cystic fibrosis patients, the univariate analysis revealed significant associations between depressive mood and female sex (OR: 4.28, 95% CI: 1.27–12.04, *p* = 0.018), as well as anemia (OR: 7.87, 95% CI: 1.50–41.27, *p* = 0.015) (Table 4). ESS also showed a significant association (OR: 1.16, 95% CI: 0.96–1.41, *p* = 0.031). No significant associations were found with age, BMI, smoking status, disease duration, hospitalization frequency, nutritional support, cardiac disease, use of oxygen support, or various sleep parameters. Similarly, no significant associations were observed regarding the pulmonary function tests, including the FEV1/L, FEV1%, FVC%, and FEV1/FVC ratios. In the multivariate analysis, female sex remained a significant predictor of depressive mood (OR: 4.12, 95% CI: 1.37–45.68, *p* = 0.026), and ESS also maintained significance (OR: 1.29, 95% CI: 0.99–1.69, *p* = 0.045). However, the association with anemia lost significance (OR: 0.22, 95% CI: 0.02–2.07, *p* = 0.184). 

## 4. Discussion

The demographic and clinical characteristics of the participants in this study revealed important insights into the relationship between depressive mood and various health parameters in bronchiectasis and CF patients. We observed that depressive mood is positive in among 28.5% of the study population between the estimated ranges when compared to the previous literature regarding chronic illnesses [17]. A significant gender difference was observed, with a higher percentage of females in the non-depressive bronchiectasis group, whereas depressive mood was more prevalent among females in the CF group (ns). This aligns with the existing literature, which often reports higher rates of depression among females with chronic illnesses [18]. In a study with 167 CF patients, Graziano et al. reported that symptoms of depression were elevated in individuals with CF and suggested that mental health should be integrated into physical healthcare for those with complex, chronic respiratory conditions [19]. Additionally, research indicates that women are more likely to experience multiple overlapping conditions, which may contribute to higher depression rates [20]. The multivariate analysis further supported the role of female sex as a significant predictor of depressive mood in CF patients. Notably, the average age and BMI were comparable between the depressive and non-depressive groups across both conditions, suggesting that these factors are not significant determinants of depressive mood in our populations. In the previous literature, these factors were reported as significant risk factors for having depression in CF [18]. Quittner et al. reported a significant difference in rates regarding age and BMI; depressive mood was observed more with advanced age and a higher BMI [21].

The modified mMRC dyspnea scale, which measures the severity of breathlessness, showed significantly higher scores in the depressive groups for both conditions. This suggests a strong association between respiratory symptom severity and depressive mood. Similar associations have been documented, highlighting the impact of severe respiratory symptoms on mental health [22,23].

Anemia emerged as a significant factor associated with depressive mood in both bronchiectasis and CF patients, particularly in the univariate analysis. This finding corroborates previous research indicating that anemia can exacerbate depressive symptoms by contributing to fatigue and reduced quality of life in chronic conditions [24,25,26]. However, this association lost significance in the multivariate analysis for CF patients, suggesting other factors may also play crucial roles. The significant associations between anemia and depression highlight the need for integrated care approaches to managing CF and bronchiectasis. Treating anemia through nutritional support and appropriate medical interventions may help reduce depressive symptoms and improve overall quality of life for these patients.

The ESS scores were significantly higher in the depressive groups, indicating greater daytime sleepiness among these patients. This aligns with research showing that poor sleep quality and excessive daytime sleepiness are common in individuals with depression and chronic respiratory diseases [27,28]. Despite this, other sleep metrics such as total sleep time, sleep efficiency, and sleep stages did not differ significantly between the groups, which could be due to the multifactorial nature of sleep disturbances in these populations.

The lung function measures showed that the depressive bronchiectasis group had a significantly lower FVC, although the diminished FVC in the depressive group of CF participants was non-significant. Reduced lung function leads to increased physical limitations and a greater disease burden, which can exacerbate depressive symptoms [29]. These results are consistent with findings from other studies where poor lung function was associated with higher levels of depression [18]. Snell et al. reported that CF patients with a lower lung capacity had higher rates of depressive mood [30].

Other clinical factors, including the prevalence of diabetes mellitus, cardiac disease, osteoporosis, and the need for oxygen support, did not show significant differences between the depressive and non-depressive groups. These findings suggest that while these comorbid conditions are important, they may not directly influence depressive mood in these populations.

Several limitations should be considered when interpreting the results of this study. Firstly, the sample size was relatively small, particularly for the cystic fibrosis subgroup, which may limit the generalizability of the findings. A larger sample size might provide more robust and definitive conclusions. Additionally, the cross-sectional design of the study prevents the determination of causal relationships between depressive mood and the various clinical and demographic factors. Longitudinal studies are needed to establish causality and examine changes over time. Furthermore, the assessment of depressive mood relied on self-reported questionnaires, which can be subject to bias and may not capture the full extent of clinical depression. Future studies could benefit from incorporating clinical interviews or diagnostic assessments to provide a more accurate measure of depressive symptoms. This study also did not control for the potential effects of medication, which could have influenced both the respiratory and psychological outcomes. Medications for respiratory conditions, as well as antidepressants, could confound the observed relationships. Detailed medication histories and their potential impact on mood and respiratory parameters should be considered in future research. Lastly, while the study included a range of clinical and demographic variables, there may be other unmeasured factors, such as socioeconomic status, lifestyle factors, and other comorbid conditions, that could influence depressive mood and were not accounted for in the analysis. Including a more comprehensive set of variables in future research could help provide a more complete understanding of the factors associated with depression in these patient populations.

## 5. Conclusions

The findings of this study underline the importance of comprehensive care approaches that address both the physical and mental health aspects of chronic respiratory disease management. Interventions aimed at improving respiratory symptoms, managing anemia, and enhancing sleep quality may help mitigate depressive symptoms in bronchiectasis and CF patients. Gender-specific strategies might also be beneficial, particularly for female patients, who appear to be at higher risk of depressive mood.

## Figures and Tables

**Table 1:** Demographics, clinical characteristics, and sleep parameters of bronchiectasis adults.

	Depressive (*n* = 20)	Non-Depressive (*n* = 50)	*p* Value
Age, years	29.65 ± 11.50	29.82 ± 11.94	0.957
BMI	22.77 ± 5.24	22.36 ± 3.78	0.750
Female sex, %	41.0	59.0	0.010
Smoker, %	15.0	12.0	0.735
Disease duration, years	16.50 ± 8.58	18.28 ± 8.30	0.434
Exacerbation count per year	1.80 ± 1.44	1.58 ± 1.33	0.542
Hospitalization count per year	0.85 ± 0.30	0.60 ± 0.15	0.411
mMRC score	1.60 ± 0.50	1.20 ± 0.45	0.004
Charlson score	0.35 ± 0.18	0.36 ± 0.09	0.459
Anemia, %	40.0	18.0	0.052
Diabetes mellitus, %	10.0	16.0	0.517
Pancreatic disease, %	40.0	40.0	1.000
Cardiac disease, %	5.0	2.0	0.496
Osteoporosis, %	0.0	6.0	0.263
O_2_ support, %	5.0	4.0	0.852
ESS	5.75 ± 3.65	3.72 ± 3.38	0.030
Sleepy, %	10.0	6.0	0.557
OSA, %	55.0	52.0	0.820
Nutrition support, %	40.0	22.7	0.126
FEV1, L	1.81 ± 0.88	2.15 ± 0.84	0.151
FEV1, %	59.15 ± 23.95	60.59 ± 22.47	0.817
FVC, L	2.40 ± 1.08	3.02 ± 0.94	0.022
FVC, %	66.67 ± 20.95	72.78 ± 20.26	0.137
FEV1/FVC	74.70 ± 12.90	70.90 ± 14.00	0.310
TST, min	351.37 ± 86.07	360.80 ± 61.37	0.659
Sleep efficiency, %	79.22 ± 13.32	80.30 ± 12.77	0.753
Sleep latency	31.33 ± 5.58	34.04 ± 5.94	0.789
WASO	57.72 ± 9.31	54.50 ± 5.81	0.769
REM latency	149.33 ± 74.07	139.84 ± 73.42	0.627
AHI	6.94 ± 5.75	6.83 ± 6.39	0.948
ODI	5.80 ± 5.36	5.74 ± 4.42	0.977
N1, min	19.90 ± 8.83	18.27 ± 12.01	0.586
N1, perc	6.03 ± 3.06	5.18 ± 3.19	0.317
N2, min	168.35 ± 57.90	173.90 ± 62.68	0.733
N2, perc	48.11 ± 11.61	49.66 ± 17.49	0.716
N3, min	104.06 ± 40.18	102.19 ± 43.29	0.868
N3, perc	29.80 ± 9.14	31.28 ± 14.34	0.670
REM, min	59.09 ± 35.42	57.45 ± 29.99	0.844
REM, perc	16.04 ± 8.40	15.62 ± 7.57	0.843

Definition of the abbreviations: AHI = apnea–hypopnea index; BMI = body mass index; ESS = Epworth Sleepiness Scale; FEV = forced expiratory volume; FVC = forced vital capacity; mMRC = Modified Medical Research Council; ODI = oxygen desaturation index; OSA = obstructive sleep apnea; REM = rapid eye movement; TST = total sleep time; WASO = wake after sleep onset.

**Table 2:** Demographics, clinical characteristics, and sleep parameters of cystic fibrosis adults.

	Depressive (*n* = 10)	Non-Depressive (*n* = 25)	*p* Value
Age, years	25.90 ± 4.41	27.04 ± 5.01	0.276
BMI	20.54 ± 3.32	21.75 ± 2.79	0.139
Female sex, %	40.0	60.0	0.084
Smoker, %	4.0	10.0	0.490
Disease duration, years	22.12 ± 5.43	17.90 ± 8.53	0.044
Exacerbation count per year	2.40 ± 1.17	1.84 ± 1.14	0.049
Hospitalization count per year	1.10 ± 0.44	0.92 ± 0.24	0.280
mMRC score	1.40 ± 0.52	1.20 ± 0.50	0.149
Charlson score	0.00	0.32 ± 0.09	0.001
Anemia, %	60.0	16.0	0.009
Diabetes mellitus, %	32.0	0.0	0.042
Pancreatic disease, %	80.0	80.0	1.000
Osteoporosis, %	0.0	12.0	0.252
O_2_ support, %	10.0	4.0	0.490
ESS	6.00 ± 3.62	3.76 ± 2.85	0.051
Sleepy, %	10.0	8.0	0.849
OSA, %	70.0	48.0	0.283
Nutrition support, %	60.0	40.0	0.283
FEV1, L	1.59 ± 0.80	1.95 ± 0.87	0.142
FEV1, %	46.44 ± 16.59	54.47 ± 23.82	0.180
FVC, L	2.25 ± 1.17	2.79 ± 1.02	0.097
FVC, %	56.55 ± 18.74	66.40 ± 22.79	0.225
FEV1/FVC	71.29 ± 9.27	70.28 ± 13.17	0.417
TST, min	358.32 ± 85.33	355.71 ± 51.86	0.457
Sleep efficiency, %	80.02 ± 14.39	78.69 ± 12.87	0.396
Sleep latency	32.44 ± 7.57	35.01 ± 10.34	0.440
WASO	56.52 ± 15.25	62.86 ± 9.11	0.358
REM latency	126.02 ± 69.91	156.62 ± 86.80	0.165
AHI	7.83 ± 4.99	6.95 ± 4.42	0.376
ODI	6.97 ± 4.48	7.12 ± 5.59	0.483
N1, min	19.15 ± 8.99	18.04 ± 12.28	0.399
N1, perc	5.65 ± 3.10	5.28 ± 3.58	0.388
N2, min	151.34 ± 54.34	162.27 ± 59.98	0.310
N2, perc	42.53 ± 10.95	46.37 ± 16.27	0.499
N3, min	117.08 ± 42.21	104.64 ± 49.25	0.244
N3, perc	33.06 ± 8.75	32.27 ± 16.52	0.444
REM, min	70.09 ± 40.61	58.38 ± 31.51	0.170
REM, perc	18.73 ± 10.01	16.06 ± 8.06	0.208
Average heart rate/min	75.57 ± 8.46	70.11 ± 8.05	0.041

Definition of the abbreviations: AHI = apnea–hypopnea index; BMI = body mass index; ESS = Epworth Sleepiness Scale; FEV = forced expiratory volume; FVC = forced vital capacity; mMRC = Modified Medical Research Council; ODI = oxygen desaturation index; OSA = obstructive sleep apnea; REM = rapid eye movement; TST = total sleep time; WASO = wake after sleep onset.

**Table 3:** Factors related to depressive mood among all bronchiectasis adults (*n* = 70).

	OR	95% CI	*p* Value
Univariate			
Age	0.99	0.99–1.04	0.956
Female sex	4.69	1.37–16.04	0.014
BMI	1.02	0.91–1.16	0.708
Smoking	1.29	0.29–5.77	0.735
Disease duration	0.97	0.91–1.04	0.420
mMRC	5.28	1.75–15.95	0.003
Exacerbation count	1.13	0.77–1.65	0.537
Hospitalization	1.20	0.78–1.86	0.408
Nutrition support	2.36	0.77–7.22	0.131
Anemia	3.04	0.96–9.86	0.048
Diabetes	0.58	0.11–3.02	0.521
Cardiac disease	2.57	0.15–43.35	0.511
Charlson score	0.98	0.47–2.10	0.958
Oxygen support	1.26	0.11–14.76	0.852
Sleepy	1.74	0.27–11.29	0.561
ESS	1.16	1.01–1.36	0.038
FEV1/L	0.61	0.31–1.20	0.153
FEV1%	0.99	0.97–1.02	0.813
FVC/L	0.49	0.27–0.93	0.028
FVC%	0.99	0.96–1.01	0.272
FEV1/FVC	1.02	0.98–1.07	0.307
Mmf%	1.00	0.98–1.02	0.835
TST	0.99	0.99–1.01	0.604
Sleep efficiency	0.99	0.95–1.03	0.749
Sleep latency	0.98	0.98–1.01	0.786
WASO	1.01	0.98–1.02	0.765
REM latency	1.00	0.99–1.01	0.622
AHI	1.03	0.92–1.09	0.947
ODI	1.00	0.94–1.07	0.977
OSA	1.13	0.39–3.19	0.820
N1 min	1.01	0.97–1.06	0.581
N1%	1.09	0.93–1.28	0.316
N2 min	0.99	0.99–1.01	0.728
N2%	0.99	0.96–1.03	0.712
N3 min	1.00	0.99–1.01	0.866
N3%	0.99	0.95–1.03	0.665
REM min	1.01	0.98–1.03	0.346
REM%	1.01	0.95–1.06	0.456
Heart rate	1.05	0.99–1.12	0.077
Multivariate			
Age	0.96	0.89–1.03	0.957
Female sex	3.27	0.57–18.79	0.183
mMRC	12.43	1.92–80.18	0.008
Anemia	3.26	0.65–16.26	0.151
ESS	1.09	0.91–1.30	0.333
FVC/L	1.05	0.44–2.50	0.910

Definition of the abbreviations: AHI = apnea–hypopnea index; BMI = body mass index; ESS = Epworth Sleepiness Scale; FEV = forced expiratory volume; FVC = forced vital capacity; mMRC = Modified Medical Research Council; ODI = oxygen desaturation index; OSA = obstructive sleep apnea; REM = rapid eye movement; TST = total sleep time; WASO = wake after sleep onset.

**Table 4:** Factors related to depressive mood among cystic fibrosis adults (*n* = 35).

	OR	95% CI	*p* Value
Univariate			
Age	0.95	0.81–1.11	0.523
Female sex	4.28	1.27–12.04	0.018
BMI	0.86	0.66–1.12	0.272
Smoking	2.66	0.15–47.03	0.504
Disease duration	0.90	0.80–1.02	0.100
mMRC	2.27	0.51–9.77	0.289
Exacerbation count	1.51	0.79–2.87	0.206
Hospitalization	1.19	0.67–2.09	0.550
Nutrition support	2.25	0.50–10.03	0.288
Anemia	7.87	1.50–41.27	0.015
Cardiac disease	2.66	0.15–47.03	0.504
Charlson score	1.01	0.98–1.02	0.512
Oxygen support	2.67	0.16–41.03	0.504
Sleepy	1.28	0.10–15.90	0.849
ESS	1.16	0.96–1.41	0.031
FEV1/L	056	0.19–1.60	0.280
FEV1%	0.98	0.95–1.02	0.352
FVC/L	0.58	0.25–1.33	0.195
FVC%	0.98	0.94–1.02	0.251
FEV1/FVC	0.98	0.93–1.03	0.513
Mmf%	0.98	0.94–1.02	0.353
TST	1.00	0.99–1.01	0.910
Sleep efficiency	1.01	0.95–1.07	0.785
Sleep latency	0.99	0.98–1.02	0.876
WASO	0.98	0.98–1.01	0.707
REM latency	0.99	0.98–1.01	0.321
AHI	1.02	0.92–1.13	0.726
ODI	0.99	0.92–1.08	0.965
OSA	2.53	0.53–12.07	0.245
N1 min	1.01	0.95–1.08	0.791
N1%	1.03	0.83–1.28	0.769
N2 min	0.99	0.98–1.01	0.610
N2%	0.98	0.93–1.03	0.488
N3 min	1.01	0.96–1.05	0.902
N3%	1.00	0.96–1.06	0.884
REM min	1.01	0.98–1.04	0.331
REM%	1.01	0.95–1.08	0.691
Heart rate	1.07	0.99–1.19	0.072
Multivariate			
Age	0.98	0.92–1.04	0.476
Female sex	4.12	1.37–45.68	0.026
Anemia	0.22	0.02–2.07	0.184
ESS	1.29	0.99–1.69	0.045

Definition of the abbreviations: AHI = apnea–hypopnea index; BMI = body mass index; ESS = Epworth Sleepiness Scale; FEV = forced expiratory volume; FVC = forced vital capacity; mMRC = Modified Medical Research Council; ODI = oxygen desaturation index; OSA = obstructive sleep apnea; REM = rapid eye movement; TST = total sleep time; WASO = wake after sleep onset.

## Data Availability

The original contributions presented in the study are included in the article, further inquiries can be directed to the corresponding author.
